# Probucol induces the generation of lipid peroxidation products in erythrocytes and plasma of male cynomolgus macaques

**DOI:** 10.3164/jcbn.18-7

**Published:** 2018-11-28

**Authors:** Mototada Shichiri, Noriko Ishida, Yoshihisa Hagihara, Yasukazu Yoshida, Aiko Kume, Hiroshi Suzuki

**Affiliations:** 1 Biomedical Research Institute, National Institute of Advanced Industrial Science and Technology (AIST), 1-8-31 Midorigaoka, Ikeda, Osaka 563-8577, Japan; 2 DBT-AIST International Laboratory for Advanced Biomedicine (DAILAB), 1-1-1 Higashi, Tsukuba-shi, Ibaraki 305-8562, Japan; 3 Health Research Institute, National Institute of Advanced Industrial Science and Technology (AIST), 2217-14 Hayashi-cho, Takamatsu, Kagawa 761-0395, Japan; 4 Research Unit for Functional Genomics, National Research Center for Protozoan Diseases, Obihiro University of Agriculture and Veterinary Medicine, Nishi 2-13, Inada-cho, Obihiro, Hokkaido 080-5555, Japan

**Keywords:** probucol, α-tocopherol, oxidative stress, anti-malarial drug, cynomolgus macaque

## Abstract

We previously reported that probucol, a lipid lowering agent, protected mice from malaria infection via depletion in plasma α-tocopherol. The antioxidant α-tocopherol in host circulation is necessary for the malaria parasites to protect themselves from oxidative stress in erythrocytes where high amounts of reactive oxygen species are generated. To assess the potential for the clinical application of probucol as an anti-malarial therapy, it was necessary to determine the effects of probucol by using primate experiments. Here we verified that probucol induces an α-tocopherol decrement in cynomolgus macaque erythrocytes and plasma. After 2 weeks of probucol administration at doses of 200 or 400 mg/kg/day, the α-tocopherol contents in erythrocytes tended to decrease. The contents of hydroxyoctadecadienoic acids and 7β-hydroxycholesterol, peroxidation products derived from linoleic acid and cholesterol, respectively, increased in erythrocytes. On the other hand, plasma α-tocopherol concentration showed a marginal decrement. Plasma lipid peroxidation products were transiently increased in the early stages of probucol administration. No adverse effects were observed throughout the experiment, although the dosage of probucol was higher than the clinical maximum dosage. Considering that malaria proliferates in erythrocytes, probucol-induced disruption of redox homeostasis in erythrocytes could be effective in the inhibition of parasite proliferation.

## Introduction

Recently, we reported that mice pre-treated with probucol, a drug used for the treatment of hyperlipidemias, were protected from malaria parasite infection.^(1,2)^ This antimalarial effect was induced by a depletion in the levels of α-tocopherol (αT), the most biologically active form of vitamin E. Probucol has been reported to inactivate the adenosine triphosphate-binding cassette transporter A1 (ABCA1)-mediated cholesterol efflux and ABCA1 is involved hepatic αT secretion into the plasma.^(3–5)^ In our previous report, plasma αT concentrations were decreased to 25% and 9% of the control levels after one day and 2 weeks of probucol treatment, respectively.^(1)^ As αT is an important antioxidant *in vivo*, αT depletion modified the redox balance. After 2 weeks treatment of probucol, the levels of linoleic acid- and cholesterol-peroxidation products in plasma were increased to 3 and 4 times that of their initial levels, respectively.^(1)^

It has been widely described that the malaria parasite is sensitive to oxidative stress.^(6–8)^ Remarkably, malaria parasites lack catalase and glutathione peroxidase, the major antioxidant enzymes in eukaryotes.^(9)^ However, they use members of the peroxiredoxin family as the principal antioxidant.^(9,10)^ During malaria infection, parasites in host erythrocytes are under conditions of high oxidative stress due to the high amount of iron in erythrocytes producing high quantities of reactive oxygen species via the Fenton reaction.^(11)^ Malaria parasites might import αT from the host and use it to protect themselves from oxidative stress.

It is possible that a drug repositioning of probucol will help the development of a new strategies for the treatment of malaria. However, in translating to the clinical use of probucol we have two problems to be assessed; the probucol effectivity on αT lowering in humans and the risk of adverse effects of high-dosage probucol. A clinical study reported that the fractional decrease of the plasma αT concentrations in hypercholesterolemic patients were 14% after three years of treatment using a standard probucol regimen, 0.5 g twice a day.^(12)^ This standard regimen of probucol is presumed not to be effective against malaria treatment. The probucol dosage used in mice (feeding with 1% containing diet) converted by the body surface area is more than 5 times that of the maximum clinical dosage used in the treatment for hyperlipidemia.^(13)^

The present study was designed to test whether probucol can reduce αT levels in primate plasma and erythrocytes and to check the adverse effects of high dosage probucol. Probucol was orally administrated to cynomolgus monkeys (*Macaca fascicularis*) and the concentrations of αT and lipid peroxidation products were measured. In this study, monkeys were not infected with the malaria parasite.

## Materials and Methods

### Ethics statement

All protocols were approved by the Committee for Experiments involving Animals of the National Institute of Advanced Industrial Science and Technology (Permit Number: 2014-218) and the Hamamatsu Pharma Research, Inc., Animal Care and Use Committee (Permit Number: HPRIRB-200).

### Monkeys

Male cynomolgus macaques (*Macaca fascicularis*) were obtained from Shin Nippon Biochemical Laboratories, Ltd. (Kagoshima, Japan) and 7- to 10-year-old macaques whose body weights were 6.1–8.0 kg were used for the study. Macaques were housed in individual stainless steel cages in light-controlled (lights on from 8:00 to 20:00) and an air-conditioned room for primates at Hamamatsu Pharma Research, Inc. Although they were housed individually, visual and auditory contacts were maintained between macaques and environmental enrichment was available in each cage. The temperature and humidity of the room were maintained at 21 ± 2°C and 60 ± 20%, respectively. Macaques were given tap water ad libitum and were fed 100 g/day of standard diet (PS-A, Oriental Yeast Co., Ltd., Tokyo, Japan) in the morning. PS-A diet (100 g) contains 14.5 mg vitamin E. The facility where this study was conducted is fully accredited by the Association for the Assessment and Accreditation of Laboratory Animal Care International.

The number of macaques for experiments used the minimum number necessary. The macaques were divided into two groups randomly so that the average body weight was equal to 7.0 kg in the 200 and 400 mg/kg/day administration groups (200 mg/kg/day group, 7.0 ± 0.8 kg; 400 mg/kg/day group, 6.9 ± 0.6 kg) (Supplemental Fig. 1A*). Humane endpoints for probucol administration were set. To determine when the animals should be euthanized, specific signs such as weight loss, loss of voluntary motion and hypothermia were examined. The health condition of the animals was monitored at least twice a day. Pain relievers or anesthesia could not be administered in this study as treatments may influence probucol metabolism.

### Administration of probucol

Probucol was obtained from Tokyo Kasei Kogyo Co. Ltd. (Tokyo, Japan). Probucol was suspended in 0.5% carboxymethyl cellulose solution at 20 or 40 mg/ml. Probucol solution (5 ml/kg body weight) was orally administered by catheter twice daily (final dosage was 200 mg/kg/day or 400 mg/kg/day). The dosage of probucol to macaques in this study was the same amount per body surface area as the amount calculated from the dose administered to mice in our previous reports.^(1)^ In our previous study, we fed mice with probucol 1% w/w in the diet. If mice weighing 25 g (84.1 cm^2^) consume a diet of 3 g/day, 30 mg of probucol is administered per day. Dividing this dose by the body surface area yields 0.357 mg/cm^2^. The dose to macaques (body weight 7 kg, body surface area 3,938 cm^2^) was 1,406 mg/day for administration at the same dose as that used for mice. By dividing this dose by the body weight, the dose was calculated to be 200 mg/kg/day. In a previous study, probucol was administered to rhesus monkeys at 0, 60, 125, 250, and 500 mg/kg/day for more than 8 years and no adverse effects were observed.^(14)^ Considering this information, the doses was set to 200 and 400 mg/kg/day in this study. The clinical dosage for humans is 500 mg (maximum dosage 1,000 mg), which is 20 mg/kg for a patient weighing 50 kg. The dosage to macaques in this study was equivalent to 10- or 20-fold the clinical dosage to humans.

### Experimental procedure

**Fig. 1:**
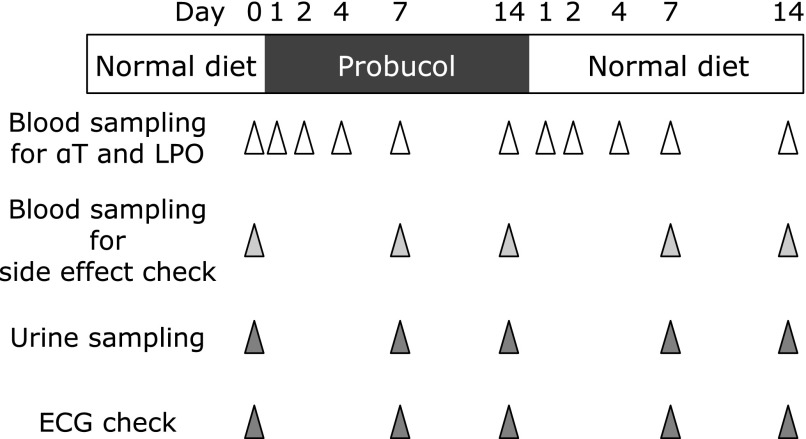
Experimental procedure for the evaluation of probucol effect. Seven- to ten-year-old male cynomolgus macaques (*Macaca fascicularis*), whose body weights were 6.1–8.0 kg, were divided into 2 groups randomly based on the dosage of probucol; 200 mg/kg/day probucol administration group (*n* = 5) and 400 mg/kg/day probucol administration group (*n* = 5). Probucol was orally administered by catheter twice daily for 2 weeks, after which probucol administration ceased. Plasma and erythrocyte samples for the analysis the concentrations of α-tocopherol and lipid peroxidation products were obtained at day 0, 1, 2, 4, 7, and 14 after starting probucol treatment and day 1, 2, 4, 7, and 14 after treatment cessation. Blood- and urine-sampling and ECG monitoring to evaluate adverse effects of probucol were obtained at day 0, 7, and 14 after starting probucol treatment and day 7 and 14 after cessation of treatment.

Macaques were divided into 2 groups by the dosage of probucol, a 200 mg/kg/day probucol administration group (*n* = 5) and a 400 mg/kg/day probucol administration group (*n* = 5). Probucol was administered for 2 weeks, after which probucol administration was ceased. Plasma and erythrocyte samples for the analysis of αT and lipid peroxidation product concentration were obtained at day 0, 1, 2, 4, 7, and 14 after starting probucol treatment and day 1, 2, 4, 7, and 14 after cessation of probucol (Fig. 1). Blood- and urine-sampling and electrocardiogram (ECG) monitoring for the evaluation of adverse effects were obtained at day 0, 7, and 14 after starting probucol treatment and day 7 and 14 after cessation of probucol (Fig. 1). Blood samples (2 ml at day 1, 2, and 4 after starting probucol treatment and day 1, 2, and 4 after cessation of probucol; 8 ml at day 0, 7, and 14 after starting probucol treatment and day 7 and 14 after cessation of probucol) were obtained from the cephalic vein. Urine samples (<1 ml) were collected from the trays under the individual cages.

### Detection of α-tocopherol by high-performance liquid chromatography-electron capture detector (HPLC-ECD)

The αT concentration in plasma and erythrocytes were measured using a protocol described previously.^(1)^ Chloroform/methanol (2/1 in volume) containing 100 µM butylated hydroxytoluene (BHT) was added to the plasma. Methanol containing 1 mM BHT was added to the erythrocyte (erythrocyte sample:methanol containing 1 mM BHT = 1:10, w/w). Lipid and vitamin E were extracted from erythrocytes and plasma by centrifugation (15,000 rpm at 4°C for 5 min). The concentrations of αT and α-tocopherylquinone were measured by using an HPLC-ECD system with an electrochemical detector (NANOSPACE SI-2, Shiseido, Tokyo, Japan) set at 700 mV, with a Wakosil-II 5C18 RS column (Wako, Tokyo, Japan) followed by a reducing column (RC-10, 15 × 4 mm; Shiseido, Tokyo, Japan). The eluent used was methanol containing 50 mM sodium perchlorate at a flow rate of 0.7 ml/min. The concentration of αT and α-tocopherylquinone were determined by comparing the area under the curve of the sample with those of the standard. The concentration of αT and α-tocopherylquinone in erythrocytes were normalized according to protein concentrations which were determined by the Bicinchoninic acid (BCA) Protein Assay Kit (Thermo Scientific, Rockford, IL).

### Measurement of high density lipoprotein

The plasma high density lipoprotein (HDL)-cholesterol concentrations were determined by using a HDL-cholesterol E-test (WAKO Pure Chemical Industries, Osaka, Japan) as previously reported.^(15)^ The process involved in this kit is the conventional phosphotungstic acid/MgCl_2_ precipitation procedure.

### Sample preparation for the analysis of lipid peroxidation products

To evaluate the levels of lipid peroxidation products, the concentrations of hydroxyoctadecadienoic acid (HODE) and 7β-hydroxycholesterol (7β-OHCh), which are oxidized from linoleic acid (LA) and cholesterol (Ch), respectively, were measured using a protocol described previously.^(1)^ Then erythrocytes and plasma were separated at 500 rpm for 5 min at 4°C. Next, erythrocytes were washed twice with a 3-fold volume of saline and extracted using a 4-fold volume of methanol containing 100 µM BHT by vortexing and centrifuging (20,400 *g* at 4°C for 10 min). The erythrocyte data were normalized to the protein concentration measured by BCA protein assay. Fifty microliters of plasma was mixed with 450 µl of saline. Subsequently, 500 µl of methanol containing the internal standards 13-HODE-d4 (5 ng) (Cayman Chemical Company, Ann Arbor, MI), 7β-OHCh-d7 (19 ng) (Medical Isotopes Inc., Pelham, NH), and 100 µM BHT, was added to 500 µl of washed erythrocytes or plasma. These were followed by reduction using 1 mM triphenylphosphine at room temperature for 30 min. The reduced samples were mixed with 1 M KOH in methanol (500 µl) under a nitrogen atmosphere and incubated on a shaker for 30 min in the dark at 40°C. The mixtures were acidified by adding 2 ml of 10% acetic acid in water and extracted with chloroform and ethyl acetate (chloroform:ethylacetate = 4:1, v/v, 5 ml). The samples were vortexed for 1 min and centrifuged at 1,750 × *g* for 10 min at 4°C. The chloroform and ethyl acetate layer was concentrated to around 1 ml divided equally into two portions for high-performance liquid chromatography with tandem mass spectrometry (LC-MS/MS) and gas chromatography-mass spectrometry (GC-MS) analysis.

### Analysis of HODE by LC-MS/MS

The divided chloroform and ethyl acetate layers were evaporated to dryness under nitrogen. The dried samples were reconstituted with methanol and water (methanol:water = 70:30, v/v, 100 µl), and portions of the samples (10 µl) were subjected to LC-MS/MS analysis. The LC consisted of an autosampler (SIL-20AC, Shimadzu, Kyoto, Japan) and a pump (LC-20AB, Shimadzu) with octadecyl-silica (ODS) column (Hypersil Gold, 3.0 µm, 100 × 2.1 mm; ThermoFisher Scientific, San Jose, CA) in a column oven (CTO-20A, Shimadzu) set at 30°C. The gradient eluent was composed of solvent A (2 mM ammonium acetate in water) and solvent B (methanol:acetonitrile = 5:95). The eluent flow rate was 0.2 ml/min. The initial gradient composition was 80% A and 20% B. This initial composition was folded for 2 min and the composition was changed to 50% A and 50% B for 45 min. Mass spectrometry was carried out using a Thermo Finnigan TSQ Quantum Discovery Max, a triple-quadrupole mass spectrometer (Thermo Fisher Scientific) fitted with an electrospray ionization source which was carried out at a needle voltage of 4.2 kV. Nitrogen was used for the sheath gas (32 psi) and auxiliary gas (10 units). The capillary was heated to 270°C, and the spectrometers were optimized to achieve the maximum sensitivity. A specific precursor-to-product ion transition was carried out by selected reaction monitoring after collision-induced dissociation in the negative mode. Argon was used as the collision gas, and the collision cell pressure was set at 1.5 mTorr. The precursor, product ions, and collision energy were determined after the optimization of MS/MS as follows: *m*/*z* = 295.0 and 194.6–195.6 at 21 eV for both 13-(*Z*,*E*)-HODE and 13-(*E*,*E*)-HODE, *m*/*z* = 295.0 and 170.5–171.5 at 24 eV for both 9-(*E*,*Z*)-HODE and 9-(*E*,*E*)-HODE, and *m*/*z* = 299.0 and 197.6–198.6 at 15 eV for 13-HODE-d4.

### Analysis of 7β-OHCh, Ch, and LA by GC-MS

The other portions of the chloroform and ethyl acetate layer were evaporated to dryness under nitrogen. A silylating agent, *N*,*O*-bis(trimethylsilyl)-trifluoroacetamide (BSTFA, 30 µl), was added to the dried samples. The solutions were vigorously mixed by vortexing for 0.5 min and incubated at 60°C for 60 min. The solutions were injected into a gas chromatograph (GC 6890 N, Agilent Technologies, Palo Alto, CA) with a quadrupole mass spectrometer (5973 Network; Agilent Technologies). A fused-silica capillary column (HP-5MS; 5% phenyl methyl siloxane, 30 m × 0.25 mm; Agilent Technologies) with helium gas as the carrier gas were used at a flow rate of 1.2 ml/min. Temperature was programmed from 60°C to 280°C at 10°C/min. The temperatures of the injector, transfer line to the mass detector, and ion source were 250°C, 250°C and 230°C, respectively. The electron energy was set at 70 eV. 7β-OHCh, Ch, and LA were identified based on their retention times and mass patterns; ions having *m*/*z* = 456 for 7β-OHCh, 458 for Ch, and 337 for LA were selected for the quantification.

### Analysis of Probucol by LC-MS

To evaluate the levels of probucol in erythrocyte and plasma were measured using LC-MS. Fifty microliters of erythrocytes were extracted using 500 µl of methanol containing 100 µM BHT containing the internal standards 13-HODE-d4 (5 ng) by vortexing and centrifuging (20,400 *g* at 4°C for 5 min). Fifty microliters of plasma was mixed with 150 µl of chloroform/methanol (2/1 in volume) containing 20 µM BHT and the internal standards 13-HODE-d4 (50 ng) by vortexing and centrifuging (20,400 *g* at 4°C for 5 min). The chloroform layer were evaporated to dryness under nitrogen and reconstituted with 500 µl of methanol. The extracted sample (10 µl) from erythrocytes and plasma were subjected to LC-MS/MS analysis. The LC consisted of a pump (LC-20AB, Shimadzu) with ODS column (Hypersil Gold, 3.0 µm, 100 × 2.1 mm; ThermoFisher Scientific) in a column oven (CTO-20A, Shimadzu) set at 40°C. The gradient eluent was composed of solvent A (2 mM ammonium acetate in water) and solvent B (methanol). The eluent flow rate was 0.32 ml/min. The initial gradient composition was 70% A and 30% B. This initial composition was folded for 2 min and the composition was changed to 6% A and 94% B for 16 min. Mass spectrometry was fitted with an electrospray ionization source which was carried out at a needle voltage of 3.6 kV. Nitrogen was used for the sheath gas (32 psi) and auxiliary gas (10 units). The capillary was heated to 270°C, and the spectrometers were optimized to achieve the maximum sensitivity. Argon was used as the collision gas, and the collision cell pressure was set at 1.5 mTorr. Probucol peak was detected at *m*/*z* = 515.

### Measurement of other parameters for evaluation of adverse effects

To evaluate adverse effects of high dose probucol we measured 13 biomarkers using blood samples. Erythrocyte-, leukocyte-, and platelet-counts in blood samples were measured by using an automatic blood cell counter (Celltac α MEK-6458, Nihon Kohden, Tokyo, Japan). Blood aspartate aminotransferase (AST), alanine aminotransferase (ALT), lactate dehydrogenase (LDH), blood urea nitrogen, and their corresponding levels in serum were determined using CicaLiquid AST, CicaLiquid ALT, CicaLiquid LDH J, CicaLiquid-N UN (Kanto Chemical Co., Inc., Tokyo, Japan), respectively. Serum creatinine levels were determined using Determiner L CRE (Kyowa Medex Co., Ltd., Tokyo, Japan). Total protein levels in serum were determined using Clinimate TP (Sekisui Medical Co., Ltd., Tokyo, Japan). Serum concentrations of sodium, potassium and chloride were measured using Bio Majesty JCA-BM8060 (JEOL, Tokyo, Japan). Plasma cortisol levels were measured using electrochemiluminescence immunoassay (ECLIA, Roche Diagnostics, Mannheim, Germany).

Urine samples were analyzed for leukocyte, urobilinogen, occult blood reaction, bilirubin, ketonic metabolite, glucose, protein and pH values by using dipstick urinalysis (Uropaper III, Eiken Chemical Co., Ltd., Tokyo).

### Electrocardiography

The ECG was recorded as described previously.^(16,17)^ Macaques received an intramuscular injection of 20 mg/kg ketamine to induce anesthesia. A 12-lead ECG was recorded for 2 min in the supine position. Electrodes for ECG were positioned on the left and right arms and the left side of the neck. The ECG waveforms (I, II, III pattern) were recorded by ECG-9422 (Nihon Kohden, Tokyo, Japan). The recording setting was 50 mm/s paper velocity, 20 mm/mV amplitude and filter cut-off 100 Hz. The waveforms were analyzed to determine RR and QT intervals. The corrected QT intervals (QTc) were calculated according to Bazett’s formula.^(18)^

### Statistics

Statistical analyses were performed using repeated measures ANOVA with Bonferroni correction by using SPSS ver. 21.0, and a *p* value of less than 0.05 was considered significant. The values of each day were compared to the initial (day 0) value the same animal. Data are presented as mean ± SE. The normality of data distribution was assessed using the Shapiro-Wilk test, and the correlations were assessed using Spearman’s exact test by using SPSS ver. 21.0.

## Results

### Probucol administration tends to reduce α-tocopherol level in erythrocytes but not in plasma

**Fig. 2:**
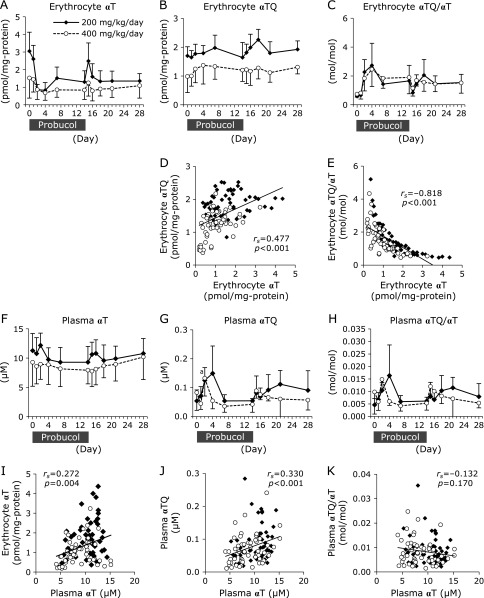
The changes in α-tocopherol (αT) and α-tocopherylquinone (αTQ) concentrations in erythrocytes and plasma. The concentrations of αT (A) and αTQ (B) in erythrocytes were measured using HPLC-ECD. The ratio of αTQ to αT (αTQ/αT) in erythrocytes was calculated (C). (D) The correlation between αT and αTQ concentrations in erythrocytes. (E) The correlation between αT level and the ratio of αTQ/αT in erythrocytes. The concentrations of αT (F) and αTQ (G) and αTQ/αT ratio (H) in plasma were analyzed. (I) The correlation between the αT concentration in plasma and that in erythrocytes. (J) The correlation between plasma αT- and αTQ-concentration. (K) The correlation between αT level and the ratio of αTQ/αT in plasma. The data are expressed as mean ± SD. Statistical analysis was carried out using ANOVA. ^a^*p*<0.05 compared to the initial (day 0) value of the same individual. To analyze the correlations, the normality of data distribution was assessed using the Shapiro-Wilk test, and the correlations were assessed by Spearman’s exact test. Solid diamonds indicate probucol 200 mg/kg/day macaque group. Open circles indicate probucol 400 mg/kg/day administered macaque group.

Probucol (200- or 400-mg/kg/day) was administered for 2 weeks. Biochemical- and physiological-analysis were conducted in accordance with schedule shown (Fig. 1). Erythrocyte αT concentrations decreased, but the decrement was not statistically significant (Fig. 2A and Supplemental Fig. 2A*). Four days after probucol administration erythrocyte αT concentrations in the 200- and 400- mg/kg/day of probucol administration group tended to decrease to 29 ± 14% and 52 ± 24% of the initial levels, respectively. Thereafter, the erythrocyte αT concentrations recovered to 46 ± 26% (200 mg/kg/day administration group) and 67 ± 39% (400 mg/kg/day administration group) of the initial levels after 14 days administration. The 1 day after cessation of probucol erythrocyte αT concentrations were transiently increased, and 14 days after cessation those of the 200- and 400-mg/kg/day administration groups were incompletely recovered (51 ± 28% and 85 ± 39% of the initial level, respectively).

The erythrocyte concentrations of α-tocopherylquinone (αTQ), the oxidation product of αT, were not notably increased in either group (Fig. 2B and Supplemental Fig. 2B*). The ratios of erythrocyte αTQ/αT were slightly increased after starting probucol administration (Fig. 2C). Four days after starting probucol administration erythrocyte αTQ/αT ratios tended to transiently increase to 482 ± 314% and 326 ± 108% of the initial level in the 200- and 400-mg/kg/day probucol administration groups, respectively (Fig. 2C). Erythrocyte αTQ levels were positively correlated with erythrocyte αT level (Fig. 2D). However, erythrocyte αTQ/αT ratios were negatively correlated with erythrocyte αT levels (Fig. 2E)

In contrast, plasma αT concentrations at 14 days after probucol administration were slightly reduced to 84 ± 17% and 92 ± 23% of the initial level in the 200- and 400-mg/kg/day groups, respectively (Fig. 2F and Supplemental Fig. 2C*). However, at 14 days after cessation complete recoveries in the plasma αT concentrations were observed in both the 200- and 400-mg/kg/day groups (98 ± 16% and 116 ± 34%, respectively). The plasma αTQ concentrations and αTQ/αT ratios were transiently increased, thereafter they decreased to almost those of the initial levels (Fig. 2G and H). Positive correlations were observed between erythrocyte αT concentration and plasma αT concentration (Fig. 2I). Plasma αT concentration was significantly correlated with plasma αTQ concentration (Fig. 2J), but not with plasma αTQ/αT ratio (Fig. 2K).

In our previous study involving probucol treatment in mice, a probucol-induced decrease in αT levels was observed in plasma but not in erythrocytes.^(1)^ The present results describing αT concentration changes in the plasma and erythrocytes of macaques are therefore contrary to those found in mice.

### Probucol effect on plasma high-density lipoprotein concentration and α-tocopherol contents

**Fig. 3:**
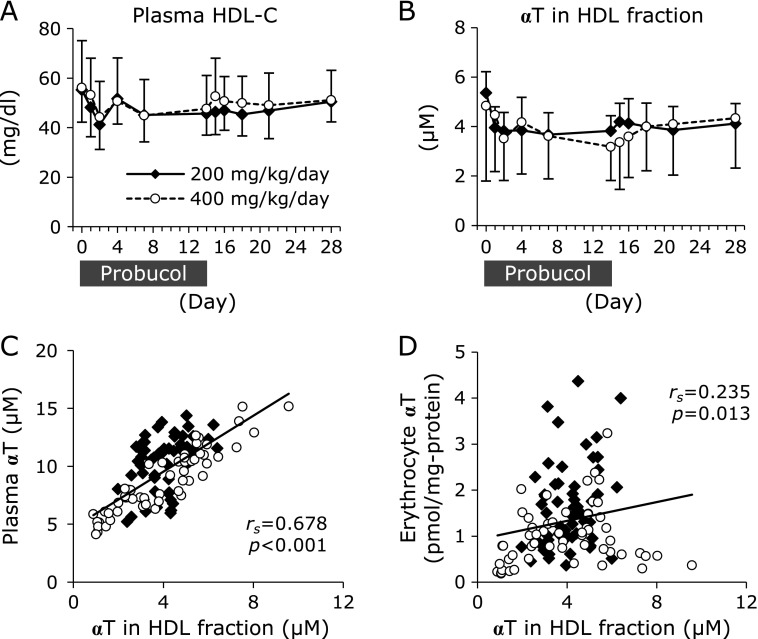
Probucol effect on plasma high-density lipoprotein concentration and α-tocopherol (αT) contents. The plasma concentrations of HDL-C (A) and αT in HDL fractions (B) were measured. The data are expressed as mean ± SD. Statistical analysis was carried out using ANOVA. (C) The correlation between the concentration of αT in HDL fractions and plasma αT concentration. (D) The correlation between the concentration of αT in HDL fractions and αT concentration in erythrocytes. The normality of data distribution was assessed using the Shapiro-Wilk test, and correlations were assessed by Spearman’s exact test. Solid diamonds indicate the probucol 200 mg/kg/day administered macaque group. Open circles indicate the probucol 400 mg/kg/day administered macaque group.

Probucol is reported to reduce the plasma concentration of high-density lipoprotein (HDL) via inactivation of the ABCA1 transporter.^(2,3)^ Furthermore, HDL has been suggested to play a role in replacing αT in erythrocytes.^(19)^ We measured the HDL-cholesterol (HDL-C) levels as this is closely related to the plasma concentration of HDL particles.^(20)^ The plasma HDL-cholesterol levels at 14 days after probucol administration were reduced to 85 ± 13% and 87 ± 10% of the initial levels in the 200- and 400-mg/kg/day groups, respectively, but the reductions were not statistically significant (Fig. 3A). Furthermore, we measured the αT contents in HDL fractions. HDL fractions were obtained from the supernatant of plasma after precipitation of apolipoprotein B-containing lipoproteins, non-HDL lipoproteins, by using phosphotungstic acid/MgCl_2_. The αT contents in HDL fractions at 14 days after probucol administration were reduced to 73 ± 13% and 81 ± 29% of the initial levels in the 200- and 400-mg/kg/day groups, respectively, but the reductions were not statistically significant (Fig. 3B). The αT content in HDL fractions was strongly correlated with plasma αT concentration (Fig. 3C). A positive correlation was observed between erythrocyte αT concentration and αT contents in HDL fraction (Fig. 3D).

### Lipid peroxidation in erythrocytes was upregulated by probucol administration

To evaluate the levels of lipid peroxidation, the concentrations of hydroxyoctadecadienoic acids (HODEs), linoleic acid (LA)-derived peroxidation products, and 7β-hydroxycholesterol (7β-OHCh), cholesterol (Ch)-derived peroxidation products, were measured. The free-radical-mediated oxidation products 9-hydroxy-10(*E*),12(*E*)-octadecadienoic acid [9-(*E*,*E*)-HODE] and 13-hydroxy-9(*E*),11(*E*)-octadecadienoic acid [13-(*E*,*E*)-HODE] were measured and the sum of both (*EE*-HODE) were calculated. 13-Hydroxy-9(*Z*),11(*E*)-octadecadienoic acid [13-(*Z*,*E*)-HODE] and 9-hydroxy-10(*E*),12(*Z*)-octadecadienoic acid [9-(*E*,*Z*)-HODE] are not only generated by free-radical-mediated oxidation but also by enzymatic oxidation via lipoxygenase. The sum of 13-(*Z*,*E*)- and 9-(*E*,*Z*)-HODE was calculated as *ZE*-HODE, and the sum of 4-isomers of HODEs was calculated as total HODE (tHODE). The stereoisomer ratio *ZE*-HODE/*EE*-HODE (*ZE*/*EE*) can be used to evaluate antioxidant capacity *in vivo*. For example, αT supplementation resulted in a high *ZE*/*EE* ratio.^(21)^

**Fig. 4:**
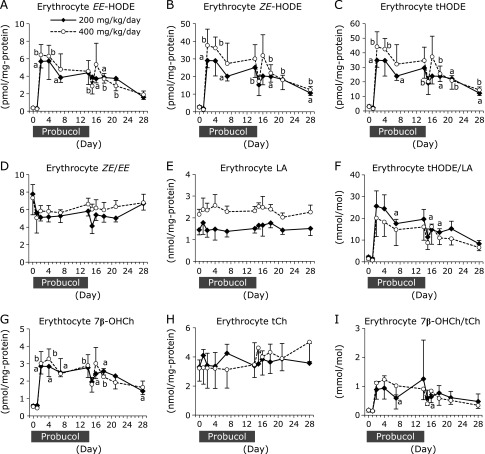
Changes in the levels of linoleic acid- and cholesterol-derived peroxidation products in erythrocytes. The erythrocyte concentrations of *EE*-HODE [the sum of 9-(*E*,*E*)-HODE and 13-(*E*,*E*)-HODE] (A), *ZE*-HODE [the sum of 13-(*Z*,*E*)- and 9-(*E*,*Z*)-HODE] (B) and total HODE (tHODE; the sum of 4-isomers of HODEs) (C) were measured by using LC-MS/MS. The ratio of *ZE*-HODE to *EE*-HODE (*ZE*/*EE*) in erythrocytes was calculated (D). The erythrocyte concentration of LA (E) was measured using GC-MS, and the ratio of tHODE in LA (tHODE/LA) was calculated (F). The erythrocyte concentrations of 7β-OHCh (G) and tCh (H) were measured by using GC-MS, and the ratio of 7β-OHCh in tCh (7β-OHCh /tCh) was calculated (I). Solid diamonds indicate the probucol 200 mg/kg/day administered macaque group. Open circles indicate the probucol 400 mg/kg/day administered macaque group. The data are expressed as mean ± SD. Statistical analysis was carried out using ANOVA. ^a^*p*<0.05 and ^b^*p*<0.05 compared to the initial (day 0) value for the same individual.

Erythrocyte content of *EE*-HODE, *ZE*-HODE, and tHODE were prominently increased after probucol administration and slowly recovered to initial levels following cessation (Fig. 4A–C). The *ZE*/*EE* ratios were decreased by probucol, but not significantly (Fig. 4D). Erythrocyte contents of LA were not changed by probucol administration (Fig. 4E), thus the tHODE/LA ratios (Fig. 4F) exhibited the same tendency as those of *EE*-HODE, *ZE*-HODE, and tHODE. Similarly, erythrocyte total Ch (tCh) contents were not changed (Fig. 4H), and erythrocyte 7β-OHCh contents and the ratios of 7β-OHCh/tCh were significantly increased after probucol administration but steadily recovered to initial levels following probucol cessation (Fig. 4G and I).

**Fig. 5:**
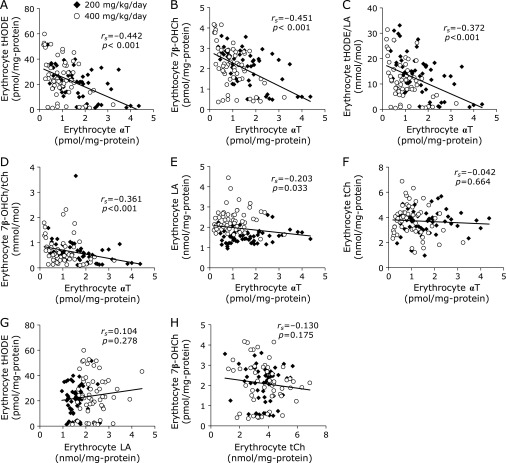
The correlation of the levels of α-tocopherol (αT) and lipid peroxidation products in erythrocytes. (A) The correlation between αT and tHODE contents in erythrocytes. (B) The correlation between αT and 7β-OHCh contents in erythrocytes. (C) The correlation between αT contents and the ratio of tHODE/LA in erythrocytes. (D) The correlation between αT contents and the ratio of 7β-OHCh/tCh in erythrocytes. (E) The correlation between αT and LA contents in erythrocytes. (F) The correlation between αT and tCh contents in erythrocytes. (G) The correlation between LA and tHODE contents in erythrocytes. (H) The correlation between tCh and 7β-OHCh contents in erythrocytes. The normality of data distribution was assessed using the Shapiro-Wilk test, and the correlations were assessed by Spearman’s exact test. Solid diamonds indicate the probucol 200 mg/kg/day administered macaque group. Open circles indicate the probucol 400 mg/kg/day administered macaque group.

Erythrocyte αT content was inversely correlated with erythrocyte tHODE content, 7β-OHCh content, tHODE/LA ratio, and 7β-OHCh/tCh ratio (Fig. 5A–D). However, the erythrocyte αT level was not correlated with erythrocyte LA and tCh contents (Fig. 5E and F), so there was no correlation between erythrocyte LA content and tHODE content, nor between the tCh content and 7β-OHCh content (Fig. 5G and H). These results indicate that the generation of lipid peroxidation products in erythrocytes was affected by erythrocyte αT content.

### Lipid peroxidation products were transiently increased in plasma by probucol administration

**Fig. 6:**
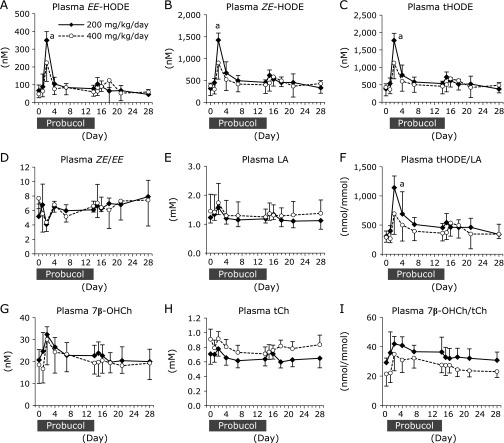
Changes in the levels of linoleic acid- and cholesterol-derived peroxidation products in plasma. The plasma concentrations of *EE*-HODE [the sum of 9-(*E*,*E*)-HODE and 13-(*E*,*E*)-HODE] (A), *ZE*-HODE [the sum of 13-(*Z*,*E*)- and 9-(*E*,*Z*)-HODE] (B) and total HODE (tHODE; the sum of 4-isomers of HODEs) (C) were measured using LC-MS/MS. The ratio of *ZE*-HODE to *EE*-HODE (*ZE*/*EE*) in plasma was calculated (D). The plasma concentrations of LA (E) was measured using GC-MS, and the ratio of tHODE in LA (tHODE/LA) was calculated (F). The plasma concentrations of 7β-OHCh (G) and tCh (H) were measured using GC-MS, and the ratio of 7β-OHCh in tCh (7β-OHCh /tCh) was calculated (I). Solid diamonds indicate the probucol 200 mg/kg/day administered macaque group. Open circles indicate the probucol 400 mg/kg/day administered macaque group. The data are expressed as mean ± SD. Statistical analysis was carried out using ANOVA. ^a^*p*<0.05 compared to the initial (day 0) value of same individual.

Plasma HODEs and 7β-OHCh concentrations were transiently increased in the early stages of the probucol administration (Fig. 6). Plasma concentrations of *EE*-HODE, *ZE*-HODE, and tHODE were significantly increased in the 200 mg/kg/day probucol group at 2 days after starting probucol administration (618 ± 286%, 490 ± 138%, 504 ± 146% of initial concentrations, respectively) (Fig. 6A–C). Thereafter, those levels were reduced after 14 days of probucol administration but are slightly higher than the initial concentrations (*EE*-HODE; 135 ± 61%, *ZE*-HODE; 161 ± 57%, tHODE; 155 ± 57% of initial concentrations in the 200 mg/kg/day group, respectively) (Fig. 6A–C). The ratio of *ZE*/*EE* was decreased at day 2 in both groups but this was not statistically significant (Fig. 6D). Plasma concentration of LA was not changed by probucol administration (Fig. 6E), thus the plasma ratio of tHODE/LA (Fig. 6F) exhibited the same tendency those of *EE*-HODE, *ZE*-HODE, and tHODE. Similarly, plasma 7β-OHCh concentration and the ratio of 7β-OHCh/tCh exhibited tendencies to increase after probucol administration (Fig. 6G and I), but were not statistically significant. Plasma tCh concentration was not changed by probucol (Fig. 6H).

**Fig. 7:**
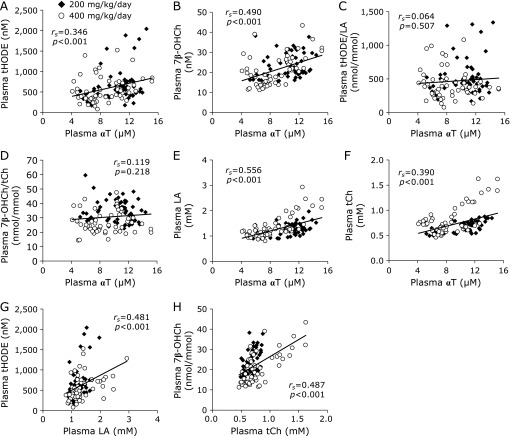
Correlation between levels of α-tocopherol (αT) and lipid peroxidation products in plasma. (A) The correlation between αT and tHODE contents in plasma. (B) The correlation between αT and 7β-OHCh contents in plasma. (C) The correlation between αT content and the ratio of tHODE/LA in plasma. (D) The correlation between αT content and the ratio of 7β-OHCh/tCh in plasma. (E) The correlation between αT and LA contents in plasma. (F) The correlation between αT and tCh contents in plasma. (G) The correlation between LA and tHODE contents in plasma. (H) The correlation between tCh and 7β-OHCh contents in plasma. The normality of data distribution was assessed using the Shapiro-Wilk test, and the correlations were assessed by Spearman’s exact test. Solid diamonds indicate the probucol 200 mg/kg/day administered macaque group. Open circles indicate the probucol 400 mg/kg/day administered macaque group.

Plasma αT concentration was positively correlated with plasma tHODE and 7β-OHCh concentrations (Fig. 7A and B) but there were no significant correlations between plasma αT concentration and plasma ratios of tHODE/LA or 7β-OHCh/tCh (Fig. 7C and D). Plasma αT concentration was also positively correlated with plasma LA and tCh concentrations (Fig. 7E and F). Further, strong correlations between plasma lipid peroxidation products and their parent lipids (Fig. 7G and H) were observed. These results indicate that the changes in plasma tHODE and 7β-OHCh concentrations were influenced by changes in plasma LA and tCh concentrations, respectively.

### Probucol concentration in erythrocytes and plasma

**Fig. 8:**
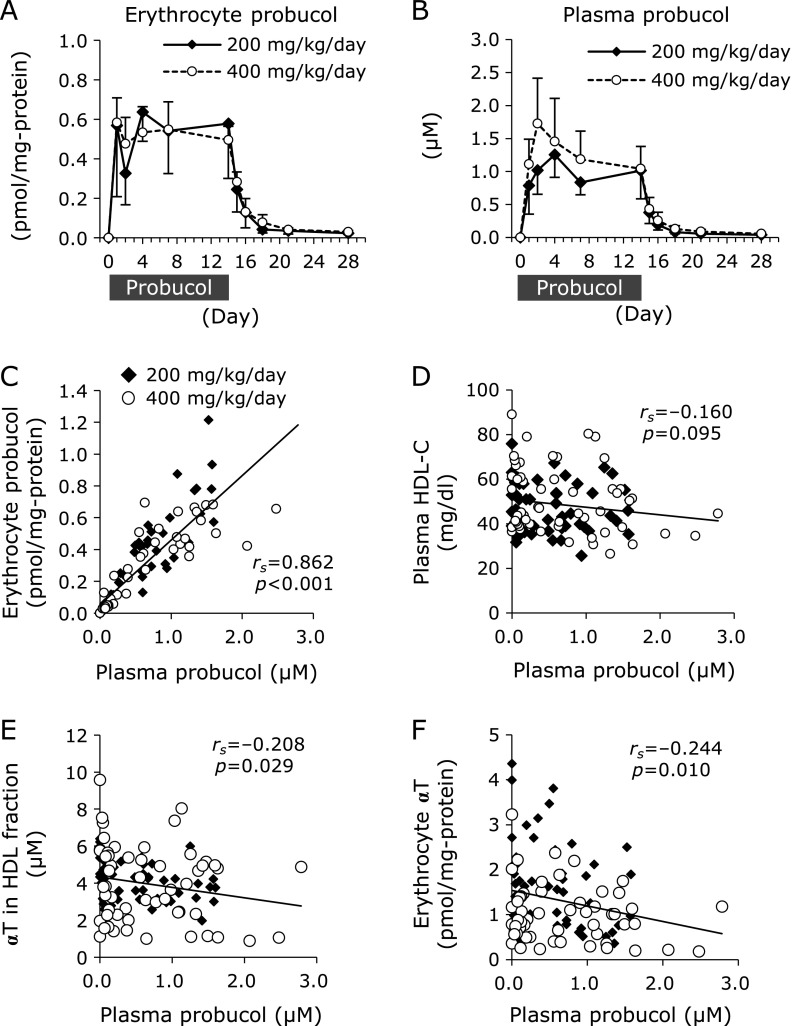
The changes in probucol concentrations in erythrocytes and plasma. The concentrations of probucol in erythrocytes (A) and in plasma (B) were measured using LC-MS. (C) The correlation between plasma probucol and erythrocyte probucol. (D) The correlation between plasm probucol and plasma HDL-C. (E) The correlation between probucol concentration in plasma and α-tocopherol (αT) concentration in HDL fractions. (F) The correlation between probucol content in plasma and αT content in erythrocytes. The data are expressed as mean ± SD. Statistical analysis was carried out using ANOVA. To analyze the correlations, the normality of data distribution was assessed using the Shapiro-Wilk test, and the correlations were assessed by Spearman’s exact test. Solid diamonds indicate probucol 200 mg/kg/day macaque group. Open circles indicate probucol 400 mg/kg/day administered macaque group.

**Fig. 9:**
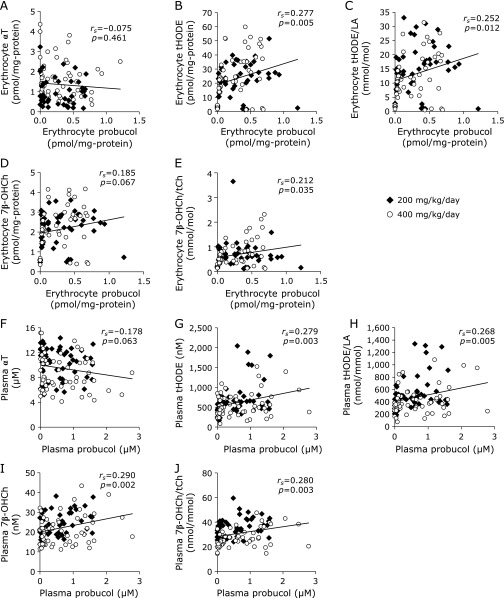
Correlation between levels of probucol and lipid peroxidation products in erythrocytes and plasma. (A) The correlation between probucol and α-tocopherol (αT) contents in erythrocytes. (B) The correlation between probucol and tHODE contents in erythrocytes. (C) The correlation between probucol content and the ratio of tHODE/LA in erythrocytes. (D) The correlation between probucol and 7β-OHCh contents in erythrocytes. (E) The correlation between probucol content and the ratio of 7β-OHCh/tCh in erythrocytes. (F) The correlation between probucol and αT contents in plasma. (G) The correlation between probucol and tHODE contents in plasma. (H) The correlation between probucol content and the ratio of tHODE/LA in plasma. (I) The correlation between probucol and 7β-OHCh contents in plasma. (J) The correlation between probucol content and the ratio of 7β-OHCh/tCh in plasma. The normality of data distribution was assessed using the Shapiro-Wilk test, and the correlations were assessed by Spearman’s exact test. Solid diamonds indicate the probucol 200 mg/kg/day administered macaque group. Open circles indicate the probucol 400 mg/kg/day administered macaque group.

The probucol concentration increased in the erythrocytes and plasma following administration of probucol (Fig. 8A and B). There was a strong correlation between the plasma probucol concentration and probucol contents in the erythrocytes (*r*_s_ = 0.862, *p*<0.001) (Fig. 8C). Regarding the relationship between the doses of probucol and the concentration of probucol in erythrocytes or plasma, there was no significant difference between the 200 mg/kg/day administration group and 400 mg/kg/day administration group (Fig. 8A and B). There was no significant correlation between probucol concentration and HDL-C concentration in the plasma (Fig. 8D). Probucol concentration in plasma was significantly correlated with αT contents in both HDL fraction (Fig. 8E) and erythrocytes (Fig. 8F). There was no correlation between the concentration of probucol and αT in erythrocytes (Fig. 9A). However, erythrocyte probucol concentration was correlated with tHODE (Fig. 9B), tHODE/LA (Fig. 9C) and 7β-OHCh/tCh (Fig. 9E) in erythrocyte. There was no significant correlation between the plasma probucol concentration and plasma αT concentration (Fig. 9F). However, plasma probucol concentration was correlated with tHODE (Fig. 9G), tHODE/LA (Fig. 9H), 7β-OHCh (Fig. 9I) and 7β-OHCh/tCh (Fig. 9J) in plasma.

### No adverse effects were caused by high-dose probucol

To evaluate the adverse effects of high dose probucol, we checked the daily general condition of the animals. No macaque exhibited listlessness, poor appetite, diarrhea, vomiting, or exanthema. Thus, the body weights in both groups of macaques did not change over the probucol administration period (Supplemental Fig. 1A*). To assess the degree of stress attributed by probucol administration, we measured plasma cortisol concentrations. The plasma cortisol concentrations of both groups were not significantly altered (Supplemental Fig. 1B*) indicating that probucol administration did not induce stress in macaques. The most important adverse effect of probucol is long QT syndrome.^(22,23)^ To assess the risk of development of long QT syndrome, we monitored the ECG and calculated the QTc according to Bazett’s formula. The mean values of QTc in both dosage groups did not significantly increase throughout the administration period or after drug cessation (Supplemental Fig. 1C*). Additionally, no animal had a QTc value elongated by over 10% of their initial QTc value (Supplemental Fig. 1D*).

The erythrocyte-, leukocyte-, and platelet counts were not significantly changed indicating that high dose probucol did not induce anemia or myelosuppressive effects (Supplemental Fig. 3A–C*). In the evaluation of hepatic function, AST was slightly decreased (Supplemental Fig. 3D*), while ALT and LDH did not change compared to the initial values (Supplemental Fig. 3E and F*). In the evaluation of renal function, there were no changes in blood urea nitrogen or creatinine (Supplemental Fig. 3G and H*). Total protein concentration was unchanged, indicating that probucol did not affect protein synthesis or renal protein reabsorption (Supplemental Fig. 3I*). Additionally, serum concentrations of sodium, chloride, and potassium were also unchanged (Supplemental Fig. 3J–L*). No animals demonstrated severe abnormal signs in urine analyses for urine leukocytes, urobilinogen, occult blood reaction, bilirubin, ketonic metabolites, glucose, protein, or pH (Supplemental Table 1*). These results indicate that high dose probucol administration did not show any adverse effects based on the markers analyzed in this study.

## Discussion

To examine the potential of probucol in the clinical setting as an anti-malarial therapy, we assessed the effect of probucol on αT levels in non-human primates. Erythrocyte αT concentrations showed a tendency to decrease although not statistically significant (Fig. 2A and Supplemental Fig. 2A*). However, a marked increase in lipid peroxidation was observed in erythrocytes (Fig. 4).

The dose-dependent effect of probucol was examined, however, there was no significant difference between the 200 mg/kg/day administration group and 400 mg/kg/day administration group for probucol contents in the plasma (Fig. 8B). ABCA1 had been reported to exist in the basolateral membrane of intestinal cells and to facilitate the absorption of cholesterol.^(24)^ It is suggested that high doses of probucol inactivate intestinal ABCA1 activity and inhibited the absorption of probucol itself, because probucol has a high degree of lipid solubility.

Regarding the relationship between the doses of probucol and αT, unexpectedly, no significant correlation was observed between the plasma probucol concentration and plasma αT concentration (Fig. 9F). The weak effect of probucol on reducing αT in the plasma (Fig. 2F) may explain the lack of a significant negative correlation between probucol and αT concentration in the plasma. In the case of mice, we had reported that probucol treatment induced a significant lowering of αT levels in plasma but not in erythrocytes.^(1)^ In contrast, the present results demonstrate that in the case of macaques the decreasing tendency of αT content was observed in erythrocytes but not in plasma (Fig. 2A and F). To consider the plasma αT concentration, the distribution of HDL among lipoprotein in circulation is an important point. HDL is the major lipoprotein in mouse plasma, comprising 67% of total lipoprotein.^(25)^ Thus, the probucol effect on αT levels was considered to be significant in mouse plasma. In contrast, the distribution of HDL in macaque’s plasma is only 11% of total lipoprotein.^(26)^ Therefore, probucol could not lower αT concentrations in macaque’s plasma significantly. In the case of primates including macaque, there may be another major transporter other than ABCA1 involved in αT efflux from the liver. For example, the ATP-binding cassette G1 (ABCG1) has been reported to be involved in cellular αT efflux.^(27)^

Furthermore, there was no correlation between the concentration of probucol and αT in erythrocytes (Fig. 9A). Jeanes *et al.*^(28)^ reported a method to determine the turnover of αT in human erythrocytes. Orally administrated ^2^H-labeled αT entered erythrocytes from plasma and pre-existing unlabeled αT was effused to plasma from erythrocytes.^(28)^ Almost 45% of αT in erythrocytes was replaced by extrinsic αT by 24 h after administration.^(28)^ The transfer of αT from HDL to erythrocytes has been demonstrated to be more efficient than other lipoproteins by using human erythrocytes, suggesting that HDL plays an important role of αT exchange in erythrocytes.^(19)^ HDL is formed by ABCA1 which function is to secrete cholesterol and phospholipids into apolipoprotein A-1.^(29,30)^ Probucol has been reported to inactivate the function of ABCA1.^(3,4)^ From these reports, we hypothesized that probucol-induced HDL deficiency shuts off the recruitment of αT to erythrocytes resulting in a lowering of αT levels and the generation of lipid peroxidation products in erythrocytes. Although plasma probucol concentration was not correlated with plasma αT (Fig. 9F) and plasma HDL-C (Fig. 8D) concentrations, probucol concentration in plasma was significantly correlated with αT contents in both HDL fraction (Fig. 8E) and erythrocytes (Fig. 8F). Considering previous reports that αT in erythrocytes is transferred from HDL,^(19,28)^ we speculated that probucol content in plasma affected αT content in HDL fraction, resulting in a decreasing tendency of αT content in erythrocytes. However, because of the low number of macaques used in this study, the decreasing tendency of αT content in HDL fraction was not statistically significant (Fig. 3B). Therefore, we consider that further experiments are necessary to clarify whether αT content in erythrocytes was affected by the decrease in αT content in HDL fraction.

Lipid peroxidation products were shown to be generated in erythrocytes. As a result of strong negative correlation between erythrocyte αT contents and the contents of lipid peroxidation products including αTQ, tHODE, and 7β-OHCh (Fig. 2E, 5A and B), the decreasing tendency of αT contents in erythrocytes is the major cause of lipid peroxidation. On the other hand, Probucol is known to have antioxidant activity.^(31–33)^ However, Gotoh *et al.*^(34)^ showed that the effect of inhibiting AMVN-induced oxidation of methyl linoleate is lower for probucol than for αT. Although probucol contents were increased in erythrocytes after probucol administration, to the values reached only 0.64 pmol/mg protein (200 mg/kg/day administration group) and 0.58 pmol/mg protein (400 mg/kg/day administration group) (Fig. 8A). These probucol concentrations increased to lower concentrations than the initial concentrations of αT, which were 3.04 pmol/mg protein in the 200 mg/kg/day administration group and 1.52 pmol/mg protein in the 400 mg/kg/day administration group (Fig. 2A). Although probucol increased in erythrocytes, the results suggest that probucol did not increase sufficiently until it could compensate for decreased αT, and thus lipid peroxidation in erythrocytes was enhanced.

In Fig. 4, *EE*-HODE and *ZE*-HODE are produced in erythrocytes. *ZE*-HODEs are formed not only by enzymatic oxidation via lipoxygenase but also by free-radical-mediated oxidation.^(35)^ *EE*-HODEs are specific products of free-radical-mediated oxidation.^(35)^ It is difficult to accurately determine what generated lipid peroxidation in erythrocytes. A previous study showed that hemoglobin in erythrocyte is a potent oxidant against lipids in the presence of hydroperoxides.^(36)^ It was suggested that there are two major routes by which heme protein species promote lipid peroxidation. First, the alkoxyl and peroxyl radicals formed during the interaction between heme species and hydroperoxides induce chain oxidation. Second, ferryl hemoglobin may take part in chain initiation. We speculated that lipid peroxidation by heme protein was enhanced by probucol-induced αT deficiency in erythrocytes.

In erythrocytes, αT concentration decreased gradually in both groups on day 1 and tended to decrease sharply on day 2 (Fig. 2A). It is thought that lipid peroxidation increased simultaneously with steep decreases in αT in erythrocytes on day 2. The mechanism by which probucol decreases αT in erythrocytes remains unclear, and the reason why αT decreased at 2 days after probucol administration is unknown. In contrast, αT in the plasma decreased only slightly on day 2 (Fig. 2F), and thus the enhancement in lipid peroxidation on day 2 cannot be explained by the decrease in αT in the plasma. Lipid peroxidation products generated in erythrocytes may have leaked into the plasma on day 2. In Fig. 4C, the tHODE content in erythrocytes was expressed as the value divided by the amount of erythrocyte protein. When the value of tHODE content in erythrocytes was expressed as a solution of erythrocytes, tHODE was 3.47 ± 1.13 µM (200 mg/kg/day group) and 3.45 ± 0.93 µM (400 mg/kg/day group) on day 0. On day 2, tHODE was increased to 41.0 ± 5.5 µM (200 mg/kg/day group) and 53.6 ± 14.1 µM (400 mg/kg/day group). In contrast, the concentration of tHODE in the plasma on day 0 was only 0.38 ± 0.14 µM and 0.42 ± 0.24 µM. Considering that the hematocrit was approximately 45%, tHODE in the erythrocytes may have been produced at a concentration 100-fold higher than that in the plasma. Additionally, reduced levels of αT in erythrocytes decreases the stability of erythrocyte membranes.^(37)^ The decreasing tendency of αT content in erythrocytes induced by probucol may have increased lipid peroxidation and instability of the erythrocyte membranes, resulting in leakage of the lipid peroxidation products from the erythrocytes into the plasma. Based on these results, lipid peroxidation products increased in the plasma on day 2, which occurred simultaneously with increased lipid peroxidation in the erythrocytes.

In conclusion, the present results showing the effects of probucol in erythrocytes are considered to be promising data for the clinical application of probucol as an anti-malarial therapy. Considering that malaria parasite proliferates in erythrocytes, probucol-induced disruption of redox homeostasis in erythrocytes could be effective in the inhibition of parasite proliferation. Furthermore, although the dosage of probucol used in this study was ten- or twenty-times higher than the maximum clinical dosage, we did not observe any adverse effects such as QT interval prolongation, which causes the potentially fatal ventricular arrhythmia “torsades de pointes.” Nevertheless, at 10 macaques, the number of primates involved in the present study was too small to confidently conclude that high dosage probucol will not increase the risk QT interval prolongation. It is necessary to confirm the adverse effects of high dosage probucol prior to clinical application. Our findings support probucol’s repositioning as an anti-malarial agent and are important to advancing future studies in the verification and validation of its clinical application.

## Author Contributions

MS: study concept and design; acquisition of data; analysis and interpretation of data; statistical analysis; drafting of the manuscript

NI: acquisition of data; analysis and interpretation of data

YH: critical revision of the manuscript for important intellectual content

YY: critical revision of the manuscript for important intellectual content

AK: critical revision of the manuscript for important intellectual content

HS: critical revision of the manuscript for important intellectual content

## Abbreviations

ABCA1: adenosine triphosphate-binding cassette transporter A1
ALT: alanine aminotransferase
αT: α-tocopherol
αTQ: α-tocopherylquinone
AST: aspartate aminotransferase
BCA: bicinchoninic acid
BHT: butylated hydroxytoluene
Ch: cholesterol
ECG: electrocardiogram
GC-MS: gas chromatography-mass spectrometry
HDL: high density lipoprotein
HODE: hydroxyoctadecadienoic acid
HPLC-ECD: high-performance liquid chromatography-electron capture detector
LA: linoleic acid
LC-MS/MS: high-performance liquid chromatography with tandem mass spectrometry
LDH: lactate dehydrogenase
OHCh: hydroxycholesterol
