# Supplementary material for: Probucol induces the generation of lipid peroxidation products in erythrocytes and plasma of male cynomolgus macaques
Source: J Clin Biochem Nutr. 2018 Nov 28;64(2):129–42. doi: 10.3164/jcbn.18-7 (PMC6436040; doi:10.3164/jcbn.18-7)
Supplement: Supplemental Figure 1 [file jcbn18-7sf01.pdf]

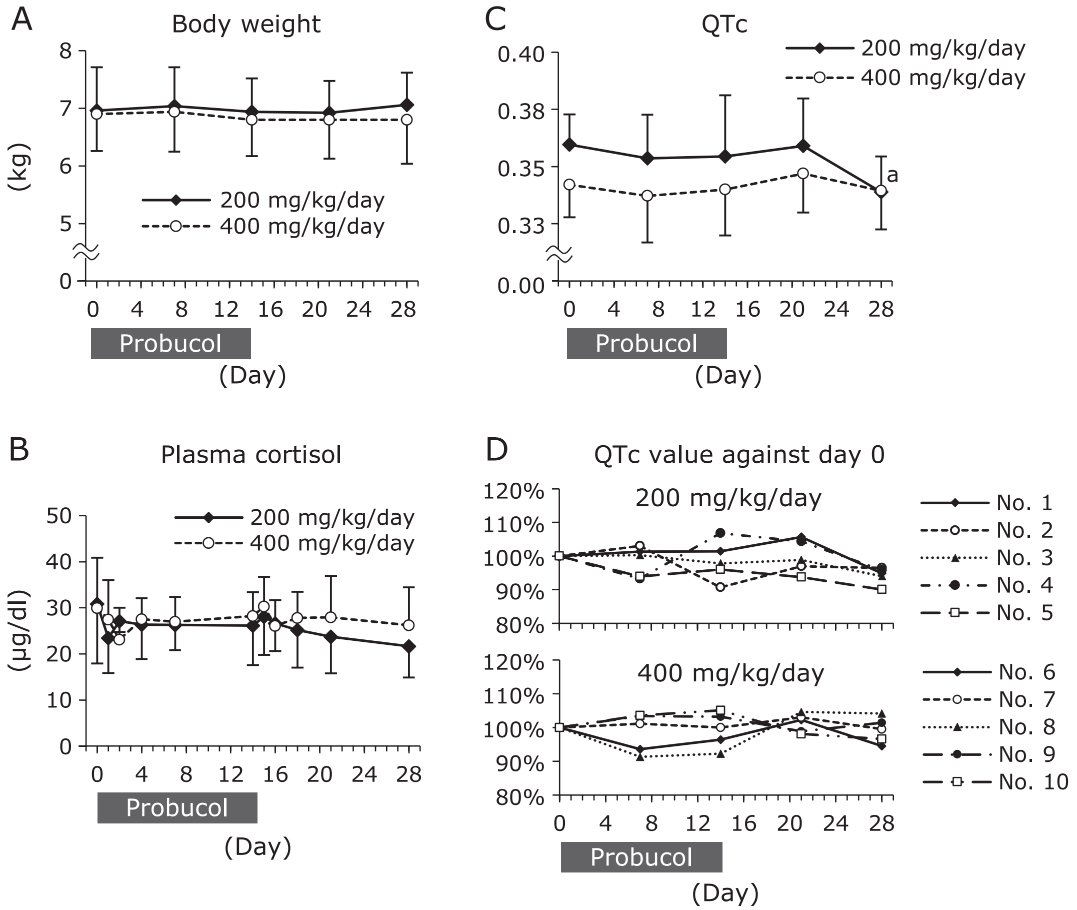

Supplemental Fig. 1. Changes in general condition and QTc on ECG. Body weights of macaques were measured at day 0, 7, and 14 after starting probucol treatment and day 7 and 14 after cessation of treatment (A). Plasma cortisol concentrations at day 0, 1, 2, 4, 7, and 14 after starting probucol treatment and at day 1, 2, 4, 7, and 14 after cessation of treatment, were measured (B). ECGs were monitored at day 0, 7, and 14 after starting probucol treatment and day 7 and 14 after cessation of treatment (C). QTc values were calculated individually (D). Solid diamonds indicate the probucol 200 mg/kg/day administered macaque group. Open circles indicate the probucol 400 mg/kg/day administered macaque group. The data are expressed as mean ± SD. Statistical analysis was carried out using ANOVA. [a]$p<0.05$ compared to the initial (day 0) value of same individual.
