# Supplementary material for: Probucol induces the generation of lipid peroxidation products in erythrocytes and plasma of male cynomolgus macaques
Source: J Clin Biochem Nutr. 2018 Nov 28;64(2):129–42. doi: 10.3164/jcbn.18-7 (PMC6436040; doi:10.3164/jcbn.18-7)
Supplement: Supplemental Figure 2 [file jcbn18-7sf02.pdf]

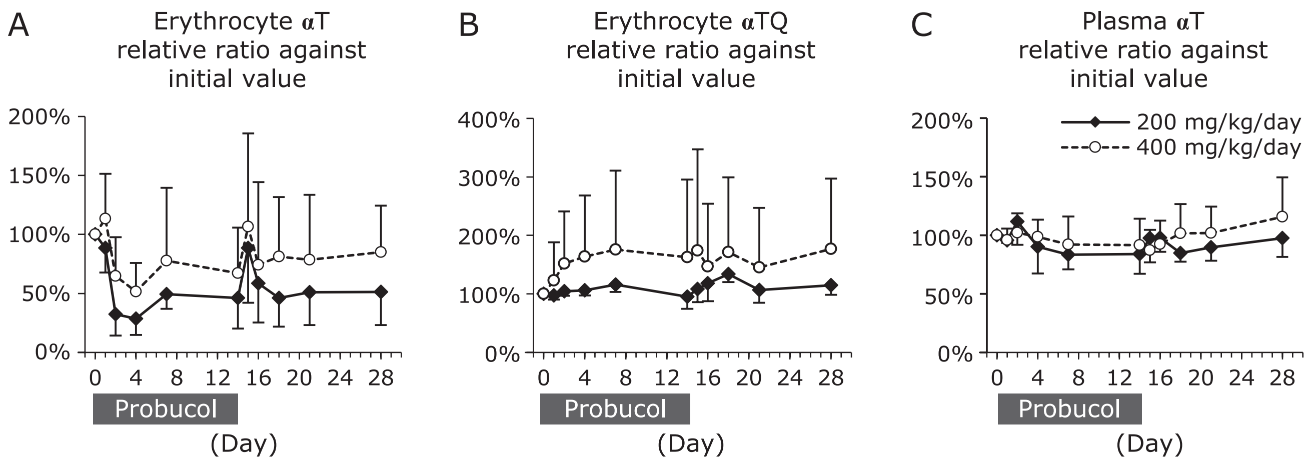

Supplemental Fig. 2. The changes from initial value of erythrocyte α-tocopherol, erythrocyte α-tocopherylquinone and plasma α-tocopherol. The average rate of change from the initial value of each monkey was shown; erythrocyte αT (A) and αTQ (B) and plasma αT (C). The data are expressed as mean ± SD. Statistical analysis was carried out using ANOVA. [a]$p<0.05$ compared to the initial (day 0) value of the same individual. Solid diamonds indicate probucol 200 mg/kg/day macaque group. Open circles indicate probucol 400 mg/kg/day administered macaque group.
