# Supplementary material for: Probucol induces the generation of lipid peroxidation products in erythrocytes and plasma of male cynomolgus macaques
Source: J Clin Biochem Nutr. 2018 Nov 28;64(2):129–42. doi: 10.3164/jcbn.18-7 (PMC6436040; doi:10.3164/jcbn.18-7)
Supplement: Supplemental Figure 3 [file jcbn18-7sf03.pdf]

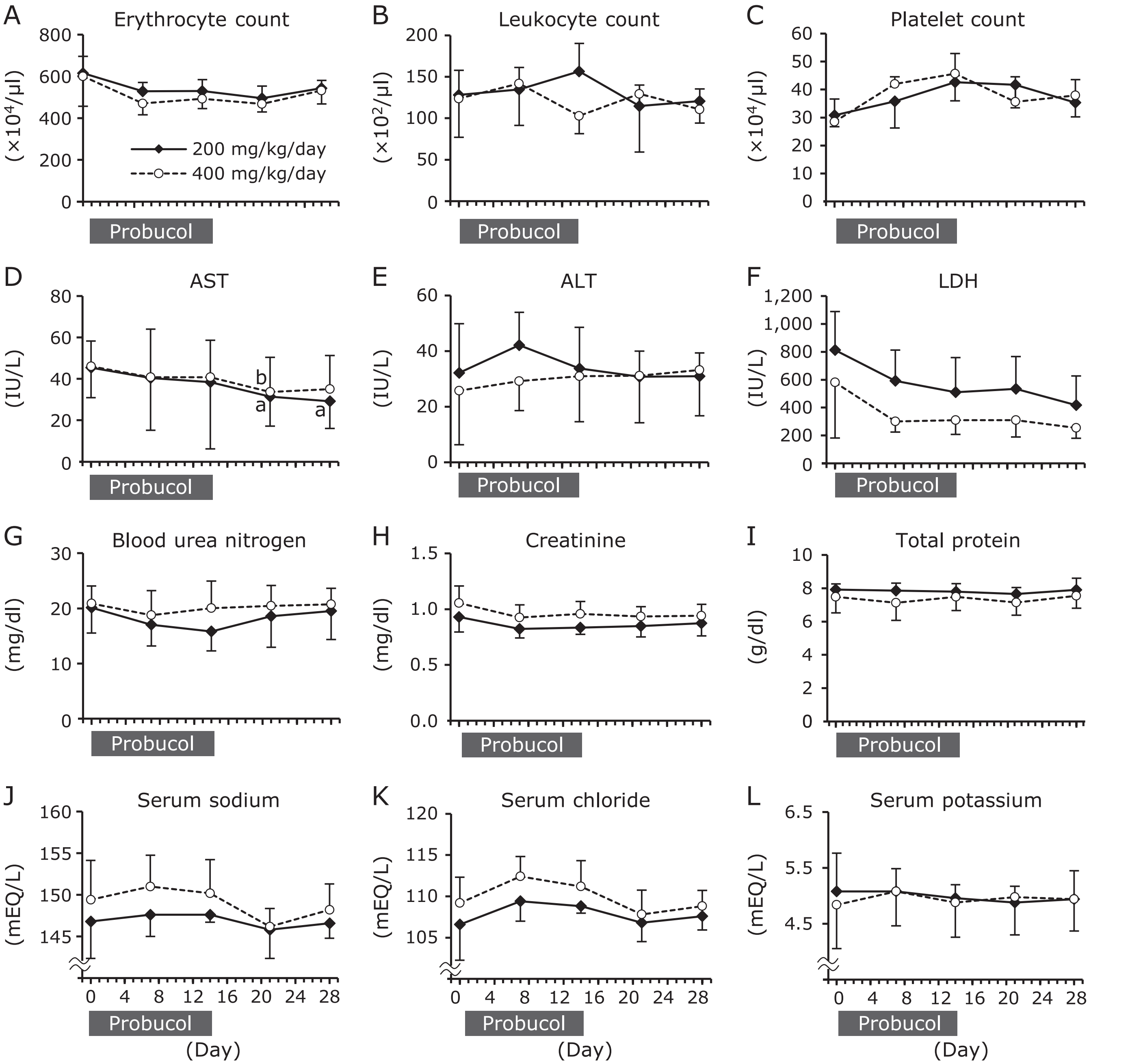

Supplemental Fig. 3. Changes in parameters for the evaluation of adverse effects. To evaluate the adverse effects of high dose probucol we measured 12 biomarkers using blood samples obtained at day 0, 7, and 14 after starting probucol treatment and day 7 and 14 after cessation of treatment. Erythrocyte count (A), leukocyte count (B), platelet count (C), aspartate aminotransferase (AST) (D), alanine creatinine aminotransferase (ALT) (E), lactate dehydrogenase (LDH) (F), blood urea nitrogen (G), creatinine (H), total protein (I), sodium (J), chloride (K), and potassium (L) were measured. Solid diamonds indicate the probucol 200 mg/kg/day administered macaque group. Open circles indicate the probucol 400 mg/kg/day administered macaque group. The data are expressed as mean ± SD. Statistical analysis was carried out using ANOVA. [a]$p<0.05$ and [b]$p<0.05$ compared to the initial (day 0) value for the same individual.
