# Supplementary material for: Probucol induces the generation of lipid peroxidation products in erythrocytes and plasma of male cynomolgus macaques
Source: J Clin Biochem Nutr. 2018 Nov 28;64(2):129–42. doi: 10.3164/jcbn.18-7 (PMC6436040; doi:10.3164/jcbn.18-7)
Supplement: Supplemental Table 1 [file jcbn18-7st01.pdf]

**Supplemental Table 1.** The results of urinalysis

| Primate | | Day | Leukocyte | | | | | Occult blood reaction | | | | | Urobilinogen | | | | | Bilirubin | | | | |
|---|---|---|---|---|---|---|---|---|---|---|---|---|---|---|---|---|---|---|---|---|---|---|
| | | Day | 0 | 7 | 14 | 21 | 28 | 0 | 7 | 14 | 21 | 28 | 0 | 7 | 14 | 21 | 28 | 0 | 7 | 14 | 21 | 28 |
| 200 mg/kg/day | No. 1 | | – | – | – | – | – | – | – | – | – | – | – | – | – | – | – | – | – | – | – | – |
| | No. 2 | | – | – | – | – | – | – | – | – | – | – | – | – | – | – | – | – | – | – | – | – |
| | No. 3 | | – | – | – | – | – | – | – | – | – | – | – | – | – | – | 1+ | – | – | – | – | – |
| | No. 4 | | – | – | – | – | – | – | – | – | – | – | – | – | – | 1+ | – | – | – | – | – | – |
| | No. 5 | | – | – | – | – | – | – | – | – | – | – | – | – | – | – | – | – | – | – | – | – |
| 400 mg/kg/day | No. 6 | | 1+ | – | – | – | – | – | – | – | – | – | – | – | – | – | – | – | – | – | – | – |
| | No. 7 | | – | – | – | – | – | – | – | – | – | – | – | – | – | – | – | – | – | – | – | – |
| | No. 8 | | – | – | – | – | – | – | – | – | – | – | – | – | – | – | – | – | – | – | – | – |
| | No. 9 | | – | – | – | – | – | – | – | – | – | – | – | – | – | – | – | – | – | – | – | – |
| | No. 10 | | – | – | – | – | – | – | – | – | – | – | – | – | – | – | – | – | – | – | – | – |

| Primate | | Day | Ketonic metabolite | | | | | Glucose | | | | | Protein | | | | | pH value | | | | |
|---|---|---|---|---|---|---|---|---|---|---|---|---|---|---|---|---|---|---|---|---|---|---|
| | | Day | 0 | 7 | 14 | 21 | 28 | 0 | 7 | 14 | 21 | 28 | 0 | 7 | 14 | 21 | 28 | 0 | 7 | 14 | 21 | 28 |
| 200 mg/kg/day | No. 1 | | – | – | – | – | – | – | – | – | – | – | – | – | – | – | – | 6 | 6 | 7 | 6 | 8 |
| | No. 2 | | – | – | – | 1+ | – | – | – | – | – | – | – | – | – | – | – | 8 | 8 | 6 | 8 | 9 |
| | No. 3 | | – | – | – | – | – | – | – | – | – | – | 1+ | – | ± | ± | – | 9 | 8 | 8 | 9 | 8 |
| | No. 4 | | 1+ | – | – | – | – | ± | – | – | – | – | ± | – | – | – | – | 7 | 7 | 6 | 7 | 6 |
| | No. 5 | | – | – | – | – | – | – | – | – | – | – | – | – | – | – | – | 9 | 9 | 7 | 8 | 9 |
| 400 mg/kg/day | No. 6 | | – | – | – | – | – | – | – | – | – | – | ± | – | ± | – | ± | 6 | 9 | 8 | 8 | 7 |
| | No. 7 | | – | – | – | – | – | – | – | – | – | – | – | – | – | – | – | 9 | 9 | 8 | 6 | 8 |
| | No. 8 | | – | – | – | – | – | – | – | – | – | – | – | ± | ± | – | – | 6 | 9 | 9 | 6 | 6 |
| | No. 9 | | – | – | – | – | – | – | – | – | – | – | – | – | – | – | – | 9 | 7 | 7 | 7 | 8 |
| | No. 10 | | – | – | – | – | – | – | – | – | – | – | – | – | – | – | – | 9 | 6 | 7 | 8 | 8 |
